# Doxorubicin Activity Is Modulated by Traditional Herbal Extracts in a 2D and 3D Multicellular Sphere Model of Leukemia

**DOI:** 10.3390/pharmaceutics15061690

**Published:** 2023-06-09

**Authors:** Laura Corzo Parada, Claudia Urueña, Efraín Leal-García, Alfonso Barreto, Ricardo Ballesteros-Ramírez, Viviana Rodríguez-Pardo, Susana Fiorentino

**Affiliations:** 1 Grupo de Inmunobiología y Biología Celular, Science Faculty, Department of Microbiology, Pontificia Universidad Javeriana, Bogotá 110231, Colombia; 2 Departamento de Ortopedia y Traumatología, Facultad de Medicina, Pontificia Universidad Javeriana, Hospital Universitario San Ignacio, Bogotá 110231, Colombia

**Keywords:** mesenchymal stromal cells, tumor microenvironment, endothelial cells, plant extracts, *Petiveria alliacea*, *Caesalpinia spinosa*, 3D multicellular cell spheroids, leukemia

## Abstract

The modulation of the tumor microenvironment by natural products may play a significant role in the response of tumor cells to chemotherapy. In this study, we evaluated the effect of extracts derived from P2Et (*Caesalpinia spinosa*) and Anamú-SC (*Petiveria alliacea*) plants, previously studied by our group, on the viability and ROS levels in the K562 cell line (Pgp− and Pgp+), endothelial cells (ECs, Eahy.926 cell line) and mesenchymal stem cells (MSC) cultured in 2D and 3D. The results show that: (a) the two botanical extracts are selective on tumor cells compared to doxorubicin (DX), (b) cytotoxicity is independent of the modulation of intracellular ROS for plant extracts, unlike DX, (c) the interaction with DX can be influenced by chemical complexity and the expression of Pgp, (d) the 3D culture shows a greater sensitivity of the tumor cells to chemotherapy, in co-treatment with the extracts. In conclusion, the effect of the extracts on the viability of leukemia cells was modified in multicellular spheroids with MSC and EC, suggesting that the in vitro evaluation of these interactions can contribute to the comprehension of the pharmacodynamics of the botanical drugs.

## 1. Introduction

Acute Leukemias (AL) have high genetic and molecular heterogeneity. Patients have low long-term survival rates (30–50%), and current therapies do not achieve complete remission [1,2,3]. Studies have shown that alterations in energy metabolism and production of reactive oxygen species (ROS) by leukemic and tumor microenvironment cells, such as endothelial cells (ECs) and mesenchymal stem cells (MSC), play an essential role in the development and progression of the leukemic process, as well as in chemoresistance [4,5,6,7]. Leukemic cells’ resistance to multiple drugs such as anthracyclines (mitoxantrone, daunorubicin, and doxorubicin) is associated with direct interaction with MSC, which favors the activation of NF-kB, c-Myc, and Notch [8,9], as well as the positive regulation of anti-apoptotic molecules [10]. The increase in ROS levels in MSC also promotes the transfer of functional mitochondria to leukemic cells, increasing up to 4.5 times the production of ATP, fatty acid oxidation, and oxidative phosphorylation (OXPHOS), which is related to chemo-resistance in the patients [10,11,12].

It has been observed that, in the leukemic niche, the expression of Nox4 in EC, as well as the production of reactive oxygen species (ROS), increase, and this is maintained even after chemotherapy with Ara-C. The authors suggest that Acute myeloid leukemia (AML) induces a hypoxic microenvironment with increased ROS, which alters the molecular signature of ECs, activating several pro-angiogenic pathways that, in vivo, may participate in tumor promotion [13]. ECs can then adopt a glycolytic metabolism that contributes to increased chemoresistance and proliferative capacity through the reverse Warburg effect [14,15,16].

Similarly, MSC increases intracellular levels of ROS, which are associated with a reduction in their osteogenic differentiation capacity, affecting normal hematopoiesis and favoring the circulation of leukemic blasts and tumor grafting in bone marrow (BM) and spleen [17,18,19,20,21].

Treatments for AL are focused on the direct elimination of tumor cells and, more recently, on the activating of the antitumor immune response [22,23]. However, other therapies, such as phytomedicines, can have a simultaneous effect on leukemic and microenvironment cells due to their multimolecular nature [24,25,26]; furthermore, in synergy with conventional therapy, they can improve the therapeutic response by reducing its toxicity and limiting the secondary effects of chemotherapy [27,28,29].

Plant-derived products such as *Tanacetum parthenium*, *Veratrum californicum*, and *Veratrum grandiflorum* have been shown to decrease relapse rates in AL patients, reducing the adverse effects of chemotherapy on the BM microenvironment [27,30]. In our group, we have been working on two plant extracts derived from *Caesalpinia spinosa* seeds (P2Et) and *Petiveria alliacea* leaves (Anamu-SC) known to have anti-inflammatory and antitumoral activity [31,32]. P2Et is a gallic acid and derivate-rich extract [33], which has high antioxidant and cytotoxic activity on several tumor cell lines. Furthermore, it has the capacity to inhibit drug resistance pumps such as Pgp, allowing it to enhance the intracellular availability of medicines such as doxorubicin [33,34,35]. Otherwise, Anamú extract is composed of several secondary metabolites, such as benzaldehyde, dibenzyl disulfide (DDS) and dibenzyl trisulfide (DTS) [36]. It can modulate glycolytic flux [31], decreasing β-F1-ATPase protein expression [32], reducing the viability of tumor cell lines [35] and leukemic blasts isolated from patients with AML and acute lymphoid leukemia (ALL) [34]. The biological activity of these extracts, which are being developed as antitumor drugs, has only been evaluated in vitro in 2D culture models.

The study of several extracts obtained from plants with anticancer potential in two-dimensional (2D) and three-dimensional (3D) culture systems allowed us to show that tumor cell response varies depending on culture type [37]. Bissel et al. demonstrated the role of the extracellular matrix in training cell behavior in the 1980s, allowing for a more precise assessment of a compound’s therapeutic potential in preclinical studies [38].

We previously demonstrated the effects of P2Et and Anamu-SC extracts on different types of tumor cells [34,39,40,41,42] and developed a 3D culture system that allowed us to evaluate the synergy between EC, MSC, and oxygen levels in the maintenance of normal hematopoietic stem cells [43,44]. With this as a backdrop, we intended to assess the effect of P2Et and Anamu-SC extracts on cell survival and ROS levels in the K562 cell line (Pgp− and Pgp+), ECs (Eahy.926), and MSC, as well as its activity in individual (2D culture) and unicellular and multicellular spheroids.

## 2. Materials and Methods

### 2.1. Plant Material

Pods of *C. spinosa* (Feuillée ex Molina) Kuntze (Divi-divi or tara) were collected in Villa de Leyva, Boyacá, Colombia and identified by Carlos Alberto Parra of the National Herbarium of Colombia (copy number of voucher COL 588448). The P2Et is produced under conditions of good manufacturing practices and chemically characterized following FDA regulations for herbal products [33,45]. Briefly, fresh pods from *C. spinosa* were dried under airflow in a solar oven at 35 °C and ground down to obtain dried plant material. Subsequently, the plant material was extracted with ethanol (96%) in a recirculating percolator. The ethanol crude extract was concentrated under vacuum and trapped on silica gel, and excess humidity was removed at 25 °C. Afterward, the ethanol extract was fractionated with ethyl acetate. P2Et extract was analyzed using UPLC analysis on an Acquity H Class UPLC Waters^®^ with Acquity photodiode array (PDA) (Milford, MA, USA) with a validated methodology. Previously, the chemical characterization of the extract was reported, where 70–95% of hydrolysable tannins can be mono-, di-, tri-O-galloyl quinic derivates calculated as 5–30% gallic acid and 2–7% methyl gallate and ethyl gallate. Three chemical standards guarantee high batch-to-batch consistency: Gallic acid, methyl gallate and ethyl gallate (Supplementary Figure S1) [33,45,46].

The leaves of *Petiveria alliacea* were collected in Quipile, Cundinamarca (Colombia), in April 2020 under the authorization of the Ministry of the Environment for the use of genetics resources through the contract access No 221 of 2018. The plant material was identified by Antonio Luis Mejia from the Colombian National Herbarium (voucher specimen number COL333406). The Anamú-SC extract was obtained and chemically characterized, as previously reported, by supercritical fluids under a standardization methodology [34]. The chemical markers used for the Anamu SC are Myricetin and dibenzyl disulfide. To guarantee batch consistency under FDA regulations for herbal drugs, the chemical profile is performed with UPLC-UV for each batch [34] (Supplementary Figure S2).

The *C. spinosa* and *P. alliacea* plant material was physically, chemically, and microbiologically certified before the production process, following the FDA Guideline (FDA *Botanical Drug Development Guidance for Industry*, 2016). In addition, the certificate of analysis for the final extract is signed under WHO regulations and USP evaluation for botanical drugs. For each test, the extracts were reconstituted with 96% ethanol.

### 2.2. Culture Conditions of Leukemic Cells and Endothelial Cells

Leukemic cell lines K562 Pgp (−) and K562 Pgp (+) were obtained as a gift from Professor Karl Tsim (HKSTU, Hong Kong) and the lines were morphologically compared with K562 (ATCCr CCL243TM, Manassas, VA, USA). Leukemic cell lines were grown in RPMI-1640 (Eurobio Scientific, Paris, France) medium supplemented with 10% fetal bovine serum (FBS) (Eurobio Scientific, Paris, France), 2 mmol/L of L-glutamine (Eurobio Scientific, Paris, France), 100 U/mL of penicillin (Eurobio Scientific, Paris, France), 100 µg/mL of streptomycin (Eurobio Scientific, Paris, France) and 25 mmol/L of Hepes (Gibco^®^, Waltham, MA, USA) at 37 °C, 5% CO_2_. The expression of Pgp protein was evaluated previously by Sandoval et al. [33], and evaluated by flow cytometry (Anti-human Pgp-FITC; clone 17F9, BD Biosciences, Franklin Lakes, NJ, USA). Briefly, 1 × 10^6^ K562 Pgp (−) and Pgp (+) cells were stained with LIVE/DEAD fixable Aqua for 20 min in dark conditions at room temperature. After washing with PBS 2% SFB, the cells were stained for 30 min at 4 °C in dark conditions with anti-human Pgp-FITC at final concentration of 1 ug/mL. The cells were acquired by flow cytometry using the BD FACS Aria U-II (BD Biosciences^®^) and the results were subsequently analyzed using FlowJo 10.8.1 Software (Tree star, Ashland, OR, USA).

Endothelial cell line Eahy.926 was donated by the Instituto de Errores Innatos del Metabolismo research group of Pontificia Universidad Javeriana. These cells were cultured in Dulbecco’s Modified Eagle’s Medium (DMEM), supplemented with 10% fetal bovine serum (FBS) and 100 U/mL of penicillin.

### 2.3. Isolation and Cultivation of MSC from Human Bone Marrow

To obtain MSC, reaming samples were collected from the intramedullary canal of the femoral head from 4 patients undergoing prosthetic hip replacement who attended the Department of Orthopedics and Traumatology of Hospital Universitario San Ignacio (Bogotá, Colombia). The sample donors participated voluntarily and accepted the informed consent previously reviewed and approved by the independent ethics committees (IECs) of the Hospital Universitario San Ignacio (Approval: 04/2020) in the ordinary session of 12 March 2020. MSCs were isolated and cultured according to protocols previously published by our group [44,47,48] and ISO 24651 international standard. MSC phenotype was confirmed by evaluating antigens as: CD34 (anti CD34-APC, clone 581, Thermo Scientific^®^, Waltham, MA, USA), CD45 (anti CD45-PerCP, clone 2D1, BD Biosciences^®^), CD73 (anti CD73-FITC, clone AD2, BD Pharmingen™, San Diego, CA, USA) and CD105 (anti CD105-PE, clone SN6, Thermo Scientific^®^) by flow cytometry using the FACSAria II-U cytometer (BD Biosciences^®^) and the data were analyzed using FlowJo 10.8.1 Software (Tree star, Ashland, OR, USA).

### 2.4. In Vitro Cytotoxicity Assays

The cytotoxic effect of each extract and doxorubicin on cell populations (K562 Pgp(−) or (+), EC and MSC) was evaluated using the MTT assay (methyl thiazole tetrazolium blue, Sigma Aldrich^®^, St. Louis, MO, USA). For this assay, cells (5 × 10^3^) were seeded in 96-well plates with P2Et (500–0 µg/mL), Anamu-SC (500–0 µg/mL) or doxorubicin (DX, 10 μM) for 48 h. Cells were also treated with ethanol (0.02%) and DMSO (0.02%), used as diluents, respectively, for plant extracts and DX solutions. For each treatment, the percentage (%) of viability was calculated according to: % viability = (Treatment absorbance/Vehicle absorbance) × 100. The IC_50_ value (50% inhibition of cell growth) was calculated using non-linear regression curves (inhibitor) versus variable slope of response plot in GraphPad Prism (GraphPad Prism 8 Software^®^, La Jolla, CA, USA).

### 2.5. Determination of Intracellular Levels of ROS in Cell Populations Treated with Plant Extracts P2Et and Anamú-SC

To evaluate intracellular ROS production in each population (K562, ECs, MSC), 2 × 10^5^ cells/mL were treated for 24 h at the IC_50_ of P2Et, Anamú-SC and DX calculated for K562 Pgp (−) and K562 Pgp (+). As control cell treatment, ethanol (0.02%) and DMSO (0.02%) were used. Cells were exposed to 1 μM of the H2DCFDA probe (2′,7′-dichlorofluorescin diacetate, Ex/Em: ~492–495/517–527 nm, D6883 Sigma-Aldrich^®^) for 40 min at 37 °C, then propidium iodide (Ex/Em: 535 nm/617 nm, Sigma-Aldrich^®^) was added for cell viability. The data were acquired in the FACSAria II-U cytometer (Becton Dickinson^®^, Franklin Lakes, NJ, USA) and analyzed with the FlowJo V 10.0 software (Tree Star, Inc^®^, Ashland, OR, USA).

### 2.6. Evaluation of the Synergistic, Additive or Antagonistic Effect of Plant Extracts with Doxorubicin in Leukemia Cells

The synergistic, additive, or antagonistic effects of the combination between P2Et and Anamú-SC extracts with DX in K562 Pgp (−) and K562 Pgp (+) leukemic cells were assessed over a dose-response matrix including eight concentrations of DX or P2Et and Anamu-SC extracts. In the K562 Pgp (−) cell line, we used DX (ranging from 0 to 1.22 μg/mL equivalent to 0–2.24 × 10^−6^ M); P2Et (ranging from 0 to 325.12 mg/mL) and for Anamu SC (ranging from 0 to 225.48 μg/mL); In the K562 Pgp(+) cell line, we used DX (ranging from 0 to 86.91 μg/mL), P2Et (ranging from 0 to 1838 mg/mL) and for Anamu SC (ranging from 0 to 2528 μg/mL). The effects of the drug combination were estimated using R the Synergy Finder Plus^®^ software and through the zero-interaction power (ZIP) model and synergy score was obtained from a dose-response matrix [49]. Interpretation score: Synergy (>10), Addition (−10 to 10) and antagonism (<10). Experiments were performed in triplicate and the results were expressed as mean ± SEM.

### 2.7. Generation of Cell Spheroids

The generation of multicellular spheroids was carried out with the following cell ratio (2(K562):2(ECs):2(MSC)) and a total cell density of 25 × 10^3^/well. The cells were seeded in 24-well plate ultra-low adherence (Corning^®^, Corning, NY, USA) with 100 μL of DMEM/F12 Advance medium (Gibco, Life Technologies^®^, Carlsbad, CA, USA), supplemented with N2 (Gibco, Life Technologies^®^) and B27 without vitamin A (Gibco, Life Technologies^®^), 100 U/mL penicillin and 100 μg/mL; then 100 μL of Matrigel Matrix Growth Factor Reduced (BD Biosciences^®^) were added to obtain a 1:1 ratio (medium:matrigel). Matrigel was solidified for 30 min at 37 °C, 5% CO_2_ and then 800 μL of supplemented DMEM/F12 Advance medium (Gibco, Life Technologies^®^) was added. This procedure was also performed for single populations (single cell-type spheroids). The cells were cultured for 10 days (37 °C and 5% CO_2_) and used for cytotoxicity assays.

### 2.8. Cytotoxicity of Plant Extracts P2Et and Anamú-SC SC on 3D Cultures (Multicellular and Single Cell-Type Spheroids)

After 10 days of culture, multicellular and single-cell spheroids were treated for 48 h with the calculated IC_50_ and sublethal concentrations of the P2Et and Anamu-SC extracts and DX (1/5 IC_50_). As a negative control, extract diluent (ethanol) and DMSO for DX were used. Spheroids were collected and centrifuged at 300× *g* for 30 s and resuspended in 100 µL of LIVE/DEAD™ Cell Imaging Kit (for green fluorescence in live cells Ex/Em: 488–515 nm; for red fluorescence in dead cells Ex/Em: 570/602 nm; Invitrogen^®^, Waltham, MA, USA) to assess cell viability through the FV1000 confocal microscope (Olympus^®^, Tokyo, Japan). Using the Olympus FV1000 confocal microscope, 640 × 640 images of 3 and 5 spheroids were acquired with a UPLFLN 10X/NA 0.3 objective and the 488 and 515 nm laser lines, and analyzed using FIJI analysis software (ImageJ, version 1.51^®^). For cytotoxicity analysis, the area from single focal plane spheroids was delimited and pixels’ area for each fluorescence channel (calcein and ethidium homodimer) was calculated. The data were analyzed and graphed using GraphPad Prism 8 software (GraphPad Software, Inc^®^, San Diego, CA, USA). Area from single focal plane spheroids was delimited.

### 2.9. Statistical Analysis

The Shapiro-Wilk test was performed to define the normality of the data. Comparisons between paired samples were performed using Mann-Whitney and Wilcoxon tests. A comparison between three or more data sets was performed using the Kruskal-Wallis test, followed by Dunn’s test comparing controls versus the experimental condition. The graphs were obtained using GraphPad Prism 8 software (GraphPad Software, Inc., San Diego, CA, USA).

## 3. Results

### 3.1. P2Et and Anamú-SC Extracts Have a Selective Cytotoxic Effect on Leukemic Cells Compared to Doxorubicin on 2D and 3D Culture

In previous studies, we have observed that both P2Et and Anamú-SC extracts have cytotoxic activity against different cell lines [35,50,51] and even against primary leukemic blasts [34]. In this work, to evaluate the effect of the tumor microenvironment recreated as a 3D culture with endothelial and mesenchymal cells on the sensitivity of the tumor lines, we begin by measuring the cytotoxic activity of the extracts against these cells to have a well-characterized model. As observed in Table 1 and Supplementary Figure S3, P2Et and Anamu-SC show a weak cytotoxic activity to mesenchymal (greater than 388 and >500 μg/mL, respectively), and endothelial cells (175 and 105 μg/mL, respectively). In contrast, doxorubicin (1.53 mM or 0.831 μg/mL and 0.34 mM or 0.184 μg/mL) was used against mesenchymal and endothelial cells, respectively, leaving a very narrow therapeutic window. This means that doxorubicin antitumor effective concentration is close to those inducing death of normal cells.

On the other hand, when the cytotoxic activity of the standardized extracts is compared, P2Et exhibits almost similar cytotoxicity in K562 Pgp(−) (81.28 μg/mL) vs. Pgp(+) (114.9 μg/mL) cells, which means that P2Et is effective both in sensitive and resistant cells, given the expression of Pgp. This had been previously reported for our group [33]. Similarly, Anamu-SC is also active in both Pgp(–) (56.37 μg/mL) and Pgp(+) (158 μ/mL), with only a few differences, compared with the preferential activity of doxorubicin to Pgp(−) cells (1.03 mM or 0.56 μg/mL), over the Pgp (+)(>18.39 mM or >10 μg/mL), by more than ten orders of magnitude. These results mean that the P2Et and Anamu-SC extracts can act on sensitive or resistant cells to chemotherapy, unlike doxorubicin, which mainly acts on sensitive cells.

**Figure 1:**
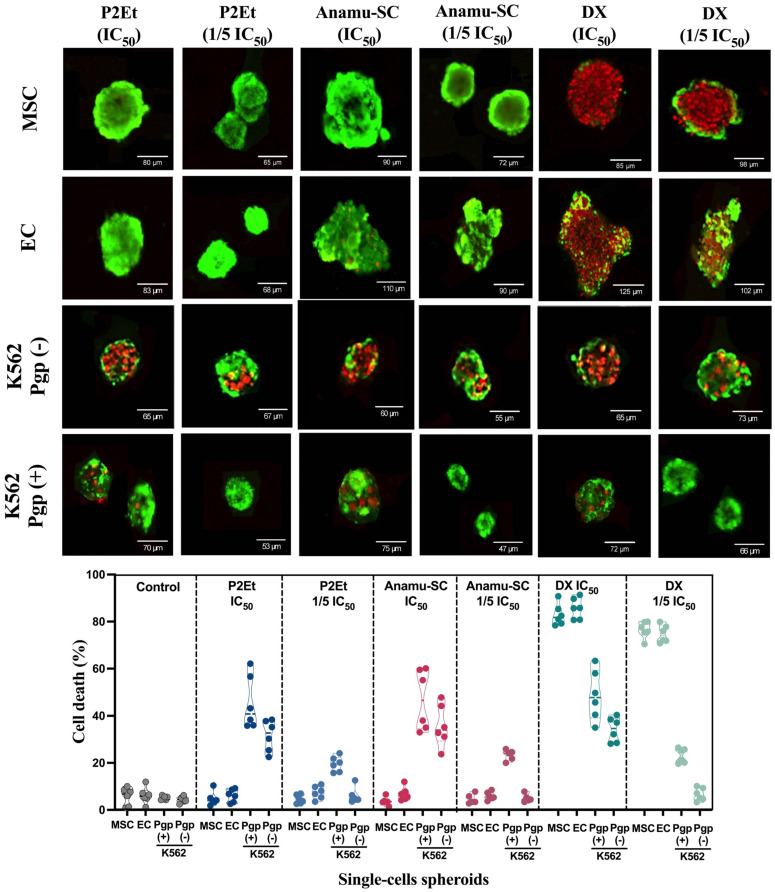
P2Et and Anamu-SC have a selective cytotoxic effect on K562 Pgp (−) single-cell spheroids. The upper figure shows a representative confocal microscopy image of the effect of P2Et (IC_50_ or 1/5 IC_50_), Anamu-SC (IC_50_ or 1/5 IC_50_) and DX (IC_50_ or 1/5 IC_50_) on the viability of single-cell spheroids derived from MSC, EC, K562 Pgp(−) or K562 Pgp(+) cells. The figure below shows the effect of P2Et and Anamu-SC extracts or doxorubicin (DX) on single-cell spheroids treated for 48 h. The extract diluent (ethanol) and doxorubicin (DMSO) were used as negative control and 100% viability was observed in all single cell-type spheres. MSC and EC spheres were treated with the IC_50_ and 1/5 IC_50_ concentrations calculated for K562 Pgp(−) and K562 Pgp(+) cells. Leukemic cell spheres were treated with the respective IC_50_. The percentage of cell death was calculated on single-cell spheroids, (*n* = 3). Viable cells: Calcein AM (green), dead cells: ethidium-1 homodimer (red). IC_50_: Inhibitory concentration 50 (drug or extract); 1/5 IC_50_: 1/5 of inhibitory concentration 50 (drug or extract).

3D cultures, such as spheroids, have been shown to modify cell behavior by mimicking in vivo conditions more closely. To evaluate whether 3D single-cell spheroids reproduce the findings of 2D cultures, we analyzed the cell death by confocal microscopy. As shown in Figure 1, P2Et and Anamú-SC did not cause any cytotoxicity on MSC or EC single-cell spheroids (Upper Figure 1, Line 1 and 2), which contrasts with the toxicity generated by DX in these cell populations (Upper Figure, Line 1 and 2). As expected, K562 Pgp (−) was more sensitive (Figure 1, Line 3) than the K562 Pgp (+) (Figure 1, Line 4). Below, Figure 1 resumes the percentage of dead cells in single-cell spheroids exposed to the extracts and DX. These results support the low toxicity of these standardized extracts, as previously observed by our group and others [34,35,50], and contribute to the explanation of secondary toxicity due to the use of conventional therapy with anthracyclines. In fact, anthracyclines induce side effects due to their activity on proliferating normal cells, inducing the appearance of mucositis or alteration in the gastrointestinal epithelium, among others.

### 3.2. Cytotoxicity Induced by P2Et and Anamu-SC Extracts Is Not Related to a Pro-Oxidant Effect

**Figure 2:**
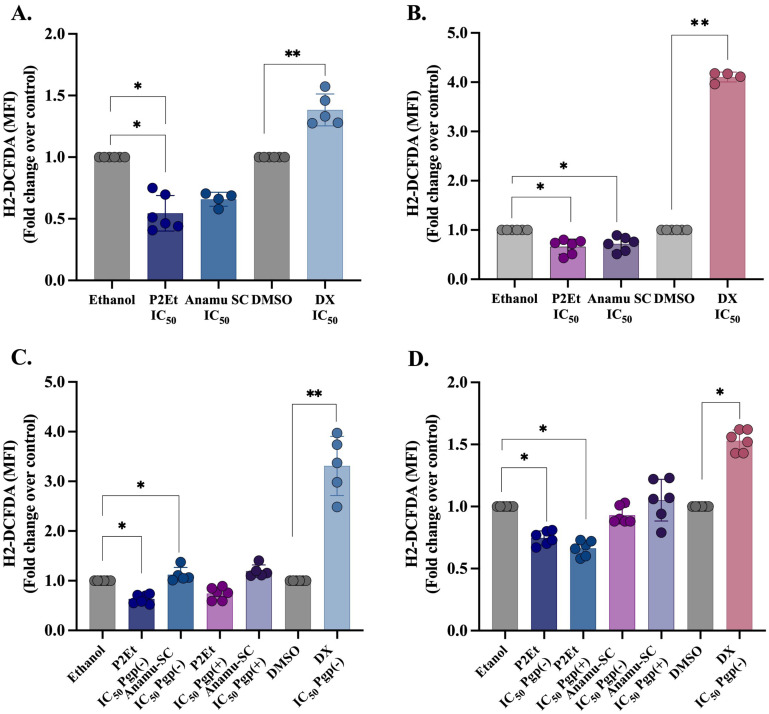
P2Et and Anamú-SC extracts have an antioxidant effect on K562 Pgp(−) and K562 Pgp(+) leukemia cells, but only P2Et has this same effect on MSC and EC. Intracellular levels of ROS were evaluated in cell populations by flow cytometry with the H2-DCFDA probe. The extract diluent (ethanol) and doxorubicin (DMSO) were used as the negative control. (**A**) K562 Pgp (−), (**B**) K562 Pgp(+), (**C**) Eahy.926-EC and (**D**) MSCs cells after individual treatment with P2Et, Anamu-SC extracts and doxorubicin (DX). (*n* = 3). * *p* < 0.05; ** *p* < 0.01.

Pro-oxidant activity has been frequently associated with the efficacy of chemotherapy. Antioxidants are not recommended while chemotherapy protocols are ongoing [52]. Alternatively, a huge antioxidant activity has been attributed to polyphenols, and natural products in general [53]. DX toxicity has been associated with increased ROS production, and additionally high ROS levels have been related to chemoresistance mediated by distinct factors, including increased Pgp expression [54]. As expected, P2Et and Anamú-SC extracts exhibit an antioxidant activity in K562 Pgp (−) and Pgp (+) cells contrasted with the pro-oxidant activity of DX (Figure 2A,B), confirming that the mechanisms involved in cell death mediated by the extracts are different from those induced by DX as has been previously shown by our group [32,35,36,41,42]. Interestingly, we observed that P2Et treatment also has an antioxidant effect on ECs and MSC (Figure 2C,D), while Anamú-SC did not modulate intracellular ROS in these cells, but no cytotoxic activity was detected with both extracts. In contrast, DX exhibits a pro-oxidant activity in both tumor and normal cells, related to its high toxicity.

### 3.3. Natural Extracts’ Interaction with DX May Depend on Chemical Complexity and Pgp Expression

Some polyphenols have been shown to act synergistically with conventional antitumor drugs [49,55,56,57]. In fact, in our group, we show that P2Et inhibits the Ppg protein, increasing the intracellular concentration of anthracyclines [33]. However, in contrast, it has been reported that Anamu-SC extract can inhibit cytochrome p450, decreasing the intracellular activation of anthracyclines and, consequently, their antitumor activity [58,59]. We evaluated the synergistic, additive, or antagonistic activity of the extracts with DX in leukemic cells (Table 2 and Supplementary Figure S4). The combination of P2Et with DX on 2D cultures showed an additive effect in K562 Pgp(−) and K562 Pgp(+) cells with a score of −8.48 + 0.51 and 0.21 + 0.83, respectively; while the interaction of Anamú-SC with DX was antagonistic in K562 Pgp (−) cells with a score of −14.88 + 1.46 and additive in K562 Pgp (+) cells with a score of −5.72 + 1.90. According to these results and considering the antioxidant effect of both extracts on K562 Pgp(+) and Pgp(−) cells, the interaction of the antioxidant compound N-acetylcysteine (NAC) with DX on these cell populations was evaluated. We show that the combination of NAC with DX presented an antagonistic effect for K562 Pgp (−) and K562 Pgp (+) with a score of −14.64 + 0.69 and −17.43 + 1.45, respectively. These results suggest that the antioxidant effect exerted by P2Et has different intracellular effects than those induced by NAC and that it does not interfere with DX cytotoxicity. Regarding Anamu-SC, the additive effect was only observed on Pgp(+) cells. Given the ability of Anamu to modulate tumor metabolism, it is necessary to study whether the expression of Pgp may be related to these results.

### 3.4. Cytotoxic Effect of P2Et and Anamú-SC Extracts Is Modified in Multicellular Spheroid, Favoring Selectivity

**Figure 3:**
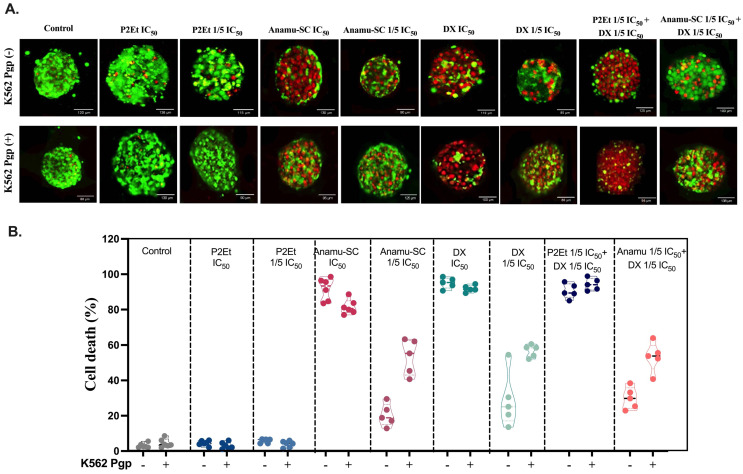
Cytotoxic effect of P2Et and Anamú-SC extracts and doxorubicin (DX) in multicellular spheroids. Multicellular spheroids were treated for 48 h with the IC_50_ and 1/5 IC_50_ of the P2Et and Anamu-SC extracts and DX calculated for K562 Pgp(−) cells and K562 Pgp(+) cells. The extract diluent (ethanol) and doxorubicin (DMSO) were used as negative control multicellular spheres. (**A**) Representative confocal microscopy images of the effect of P2Et, Anamu-SC and DX in individual treatment, and in combination of P2Et or Anamu-SC with DX on the viability of multicellular spheroids. Viable cells: Calcein AM (green), dead cells: ethidium-1 homodimer (red). (*n* = 3). (**B**) Percentage of cell death was calculated on the multicellular spheroids evaluated by confocal microscopy, the data are presented as violin plots and each point represents an independent sample. (*n* = 3). IC_50_: Inhibitory concentration 50 (drug or extract); 1/5 IC_50_: 1/5 of inhibitory concentration 50 (drug or extract).

Multicellular spheroids, composed of tumor cells co-cultured with EC and MSC, allow us to assess whether this microenvironment modifies the response to DX and/or extracts. As observed on Figure 3, DX and Anamu-SC exhibit an important cytotoxic activity both on K562 Pgp (−) and Pgp (+) cells, contrary to P2Et, which do not have any effect on the multicellular spheroids. When extracts and DX are put together, co-treatment with P2Et at sublethal doses of both the extract and the DX increases the death of Pgp (−) tumor cells by 3.6 times and that of Pgp (+) by 1.5 times. Given the result obtained on the unicellular spheres, where it was shown that P2Et had no cytotoxic effect on the EC and MSC spheres, but on tumor cells (Figure 1), it can be assumed that the death observed in the multicellular spheres is of the tumor cells. Moreover, the low sensitivity of multicellular spheroids to P2Et might be explained because of the interaction with EC and MSC, which can protect tumor cells from the extract activity. In addition, interestingly, the synergy between P2Et and sublethal concentrations of DX could favor the use of lower concentrations of this chemotherapy, thus reducing its side effects observed in leukemia patients [60]. It is possible that additive activity of P2Et is not due to its antioxidant effect but to other phenomena related to the induction of collateral sensitivity [61,62]. Contrary, Anamu-SC exhibits an important cytotoxic activity on the multicellular spheroids of both K562 Pgp(−) and (+) cells, alone in a dose-dependent manner and combined with a sublethal concentration of DX and does not reduce DX cytotoxicity, suggesting that the Anamu-SC extract by itself could be used, since it presents antitumor activity even in the presence of EC and MSC, but does not present cytotoxicity against these cells.

## 4. Discussion

Phytotherapy has been widely used to improve the response to conventional treatment in distinct types of cancer, generating positive effects on patients’ survival and quality of life [31,39,63] through direct and indirect beneficial effects [25]. In this study, we evidenced that P2Et and Anamú-SC plant extracts are not toxic to microenvironment BM cells such as ECs and MSC, regardless of the culture system evaluated (2D vs. 3D), as we had previously shown for other cell populations, such as fibroblasts and peripheral blood mononuclear cells [32,33,35]. In addition, plant extracts have been reported to exert their antitumor effect with lower doses than conventional chemotherapeutics [64].

Contrary to the effect on normal cell populations, P2Et and Anamu-SC extracts have a selective cytotoxic effect mainly on K562 Pgp(−) cells compared to Pgp(+), both in single cell-type spheroids and in 2D cultures, with all treatments and independent of ROS modulation. Interestingly, the cytotoxic effect of the P2Et extract was modified when tested on leukemic cells cocultured with ECs and MSC in multicellular spheroids. Whereas the P2Et extract was cytotoxic only when combined with sublethal concentrations of doxorubicin regardless of Pgp protein expression and ROS levels, the cytotoxicity of the Anamu-SC extract or DX was not changed when combined.

In contrast, the cytotoxicity of Anamu-SC does not change in 3D culture. We were also able to observe that, using 1/5 of the IC_50_ of DX, no significant cytotoxic activity was observed, but when cells are treated with 1/5 of the IC_50_ of DX together with 1/5 of the IC_50_ of P2Et, the cytotoxic activity suggests that small concentrations of the extract have a biological effect. The synergistic activity of P2Et can be explained by the inhibition of the Pgp pump in Pgp(+) cells, as we have previously reported [33], but also by collateral sensitivity mechanisms that could be exerted by the extract and that would be evident in Pgp(−) cells [61,62].

The results obtained with Anamu-SC are interesting. This extract, contain some metabolites such as dibenzyl disulfide (DDS), that can inhibit cytochrome P450 enzymes at high concentrations [59], reducing the intracellular processing of doxorubicin and hence its biological activity. In this study, we observed that the interaction of Anamu SC with doxorubicin is antagonistic in 2D culture for K562 Pgp(−) tumor cells but not for Pgp(+). Interestingly, these differences are not the same when the activity in the multicellular spheres is analyzed, in which it is observed that both Anamu SC and DX exert a cytotoxic activity on the spheres, this being the same when the two compounds are used together.

The use of 3D models has been widely explored to study the activity of natural products isolated or still present in botanical extracts [65,66] and there is undoubtedly a growing interest in the use of these 3D platforms in drug discovery [67] or in the study of the mechanisms regulating epithelium–mesenchymal transition [68]. Recently, Fonseca-Benitez et al. [69] showed that an extract of *Passiflora mollissima* can inhibit the growth of oral cancer cells cultured in tumor spheroids, through the increase of p53, HIF1a and CDH1.

In fact, in the search to improve the quality of life of cancer patients, integrative medicine has addressed the development of models that allow understanding of how natural products can have protective or even antitumor activity. As a contribution to this, Robert and collaborators have developed a high-throughput imaging strategy to investigate the responses of 3D HCT116 spheroids to some phytochemicals present in food [70]. Although this strategy, as well as others in which monocellular spheroids are used [71], has led to progress in the knowledge of natural products activity, there are not many hetero-cellular models with which to study the leukemic niche and fewer to assess the role of botanical natural products. In this study, we demonstrate that the cytotoxic effect of plant-derived extracts with potential anti-leukemic effects can be modified if evaluated in 3D culture models where other cell populations residing in the leukemic niche are involved. Given the complexity of the leukemic niche, it is necessary to carry out pre-clinical evaluations in systems that more closely mimic the medullary microenvironment and even more so when it comes to botanical extracts.

## 5. Conclusions

In this work, we demonstrate that the plant-derived extracts P2Et and Anamu-SC are not toxic to resident cell populations of the bone marrow but have a toxic effect directed at leukemic cells, which adds value to their potential as an adjuvant in antileukemic therapy without increasing the effects associated with conventional chemotherapy.

The results obtained separately on endothelial and mesenchymal cells in 2D and 3D cultures led us to hypothesize that cell death generated by the extract, within the spheroid is directed against tumor cells and not against the other cell types. P2Et only has cytotoxic activity in 3D cultures when multicellular spheroids are co-treated with doxorubicin. This may support its activity as an adjuvant and protector of the tumor microenvironment without reducing the cytotoxic activity of chemotherapy. In contrast, Anamú’s antileukemic activity is evident even when it is used as a single treatment, without affecting the integrity of endothelial or mesenchymal cells.

## Figures and Tables

**Table 1:** IC_50_ values of Doxorubicin (DX), P2Et and Anamu-SC extract in mesenchymal stromal cells (MSC), endothelial cells (EC), Leukemia cells (K562 Pgp(−) or K562 (+)) in standard culture conditions (2D).

Cell Line	Doxorubicin (DX) (μM)	P2Et (μg/mL)	Anamu-SC (μg/mL)
MSC	1.53 ± 0.128	388.81 ± 9.46	>500 ± 10.1
EC	0.34 ± 0.101	175.0 ± 4.33	105.5 ± 11.8
K562 Pgp (−)	0.56 ± 0.018	81.28 ± 12.2	56.3 ± 8.37
K562 Pgp (+)	>10 ± 9.53	114.9 ± 5.88	158 ± 2.53

**Table 2:** Interaction between P2Et and Anamu-SC extracts, N-aceltylcisteine (NAC) and doxorubicin (DX).

Cell Line	Combination	ZIP Score	Interpretation
K562 Pgp(−)	P2Et + DX	−8.94 ± 0.51	Addition
	Anamu-SC + DX	−14.88 ± 1.46	Antagonism
	NAC + DX	−14.69 ± 0.69	Antagonism
K562 Pgp (+)	P2Et + DX	0.21 ± 0.83	Addition
	Anamu-SC + DX	−5.72 ± 1.90	Addition
	NAC + DX	−17.43 ± 1.45	Antagonism

Abbreviations: DX (Doxorubicin), NAC (N-acetylcysteine); Interpretation score: Synergy > 10, Addition −10 to 10, Antagonism < 10.

## Data Availability

The data that support the findings of this study are available from the corresponding author upon reasonable request.

## Supplementary Materials

The following supporting information can be downloaded at: https://www.mdpi.com/article/10.3390/pharmaceutics15061690/s1, Figure S1: UPLC-PDA chromatogram at 274 nm of P2Et extract. Peak Identification: (1), Gallic acid; (2), Methyl gallate; (3), Ethyl Gallate; Figure S2: UPLC-PDA chromatogram at 274 nm of Anamu SC. Peak Identification: (1), myricetin; (2), dibenzyl disulfide; Figure S3: P2Et and Anamu-SC have a selective cytotoxic effect on K562 Pgp(−) in 2D cultured; Figure S4: Effect of the combination of the P2Et and Anamu-SC extracts with doxorubicin (DX) in leukemic cells.
