# Supplementary material for: Doxorubicin Activity Is Modulated by Traditional Herbal Extracts in a 2D and 3D Multicellular Sphere Model of Leukemia
Source: Pharmaceutics. 2023 Jun 9;15(6):1690. doi: 10.3390/pharmaceutics15061690 (PMC10302921; doi:10.3390/pharmaceutics15061690)
Supplement: Supplementary file 1 [file pharmaceutics-15-01690-s001.zip › pharmaceutics-2377922-supplementary.pdf]

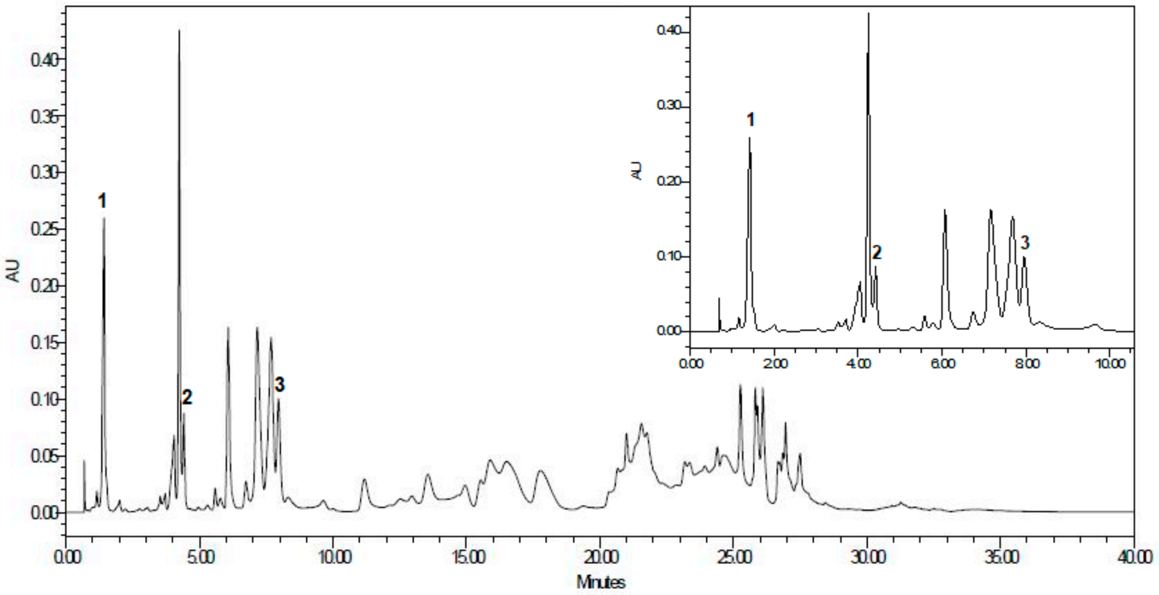

Figure S1. UPLC-PDA chromatogram at 274 nm of P2Et extract. Peak Identification: (1), Gallic acid; (2), Methyl gallate; (3), Ethyl Gallate

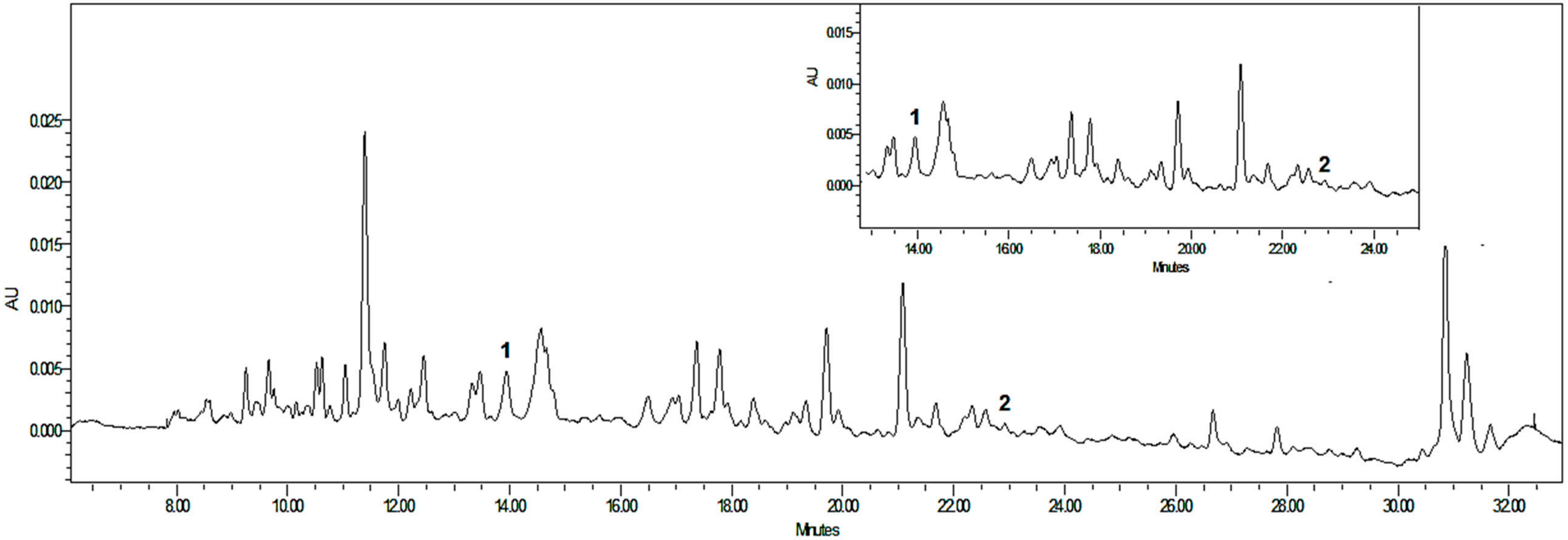

Figure S2. UPLC-PDA chromatogram at 274 nm of Anamu SC. Peak Identification: (1), myricetin; (2), dibenzyl disulfide.

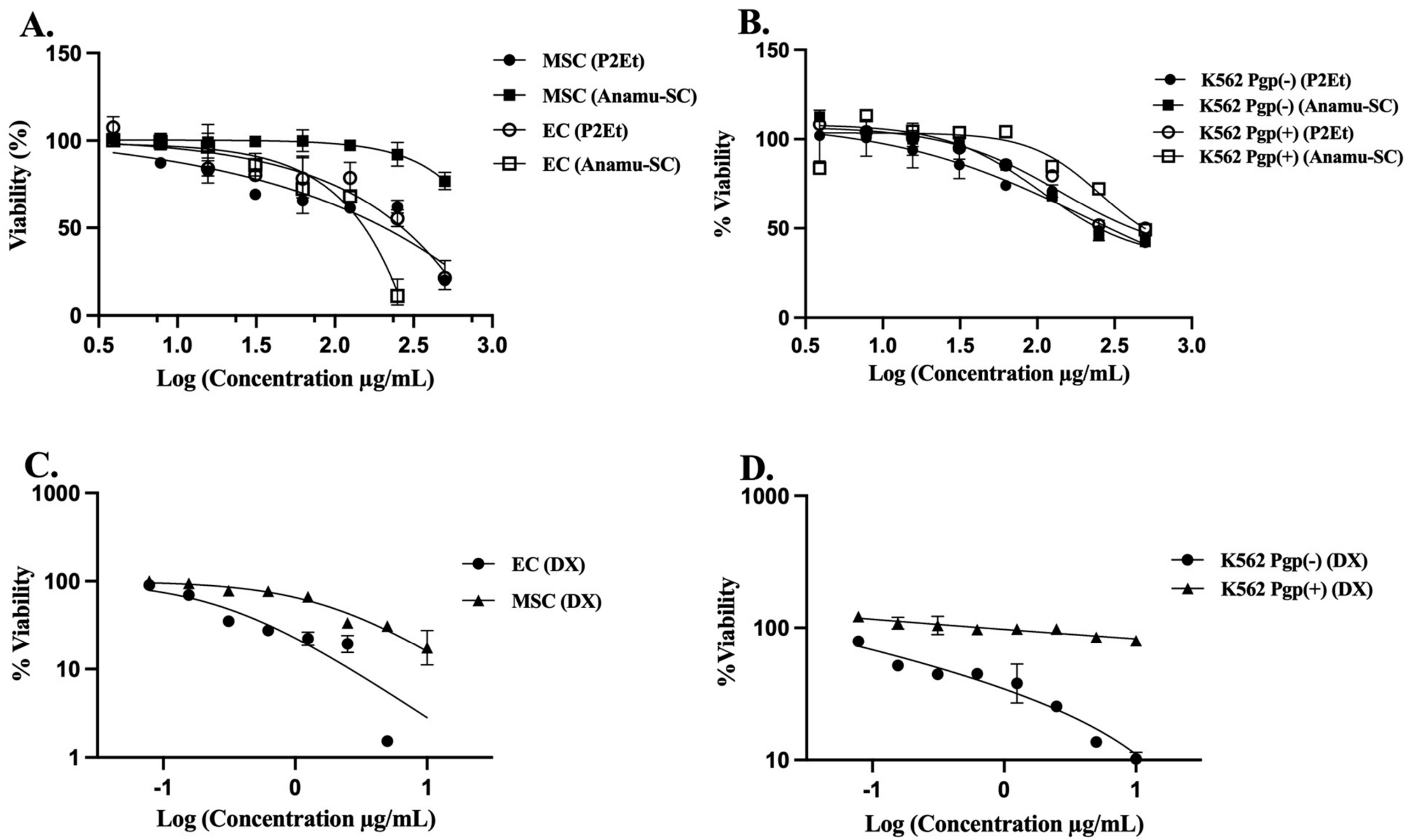

Figure S3. P2Et and Anamu-SC have a selective cytotoxic effect on K562 Pgp(-) in 2D cultured. A. Mesenchymal cells (MSC) and Endothelial cells (EC) were treated for 48 hours with serial dilutions of P2Et (filled circles) and Anamú-SC (filled square) extracts or P2Et (empty circles) and Anamú-SC (empty square). **B.** K562 Pgp(-) and K562 Pgp (+) cells were treated for 48 hours with serial dilutions of P2Et (filled circles) and Anamú-SC (filled square) extracts or P2Et (empty circles) and Anamú-SC (empty square).(**C**) EC or MSC cells were treated for 48h with doxorubicin (DX) (filled circle) or (filled triangle) respectively. (**D**) K562 Pgp(-) and K562 Pgp (+) cells were treated for 48h with doxorubicin (DX) (filled circle) or (filled triangle) respectively.

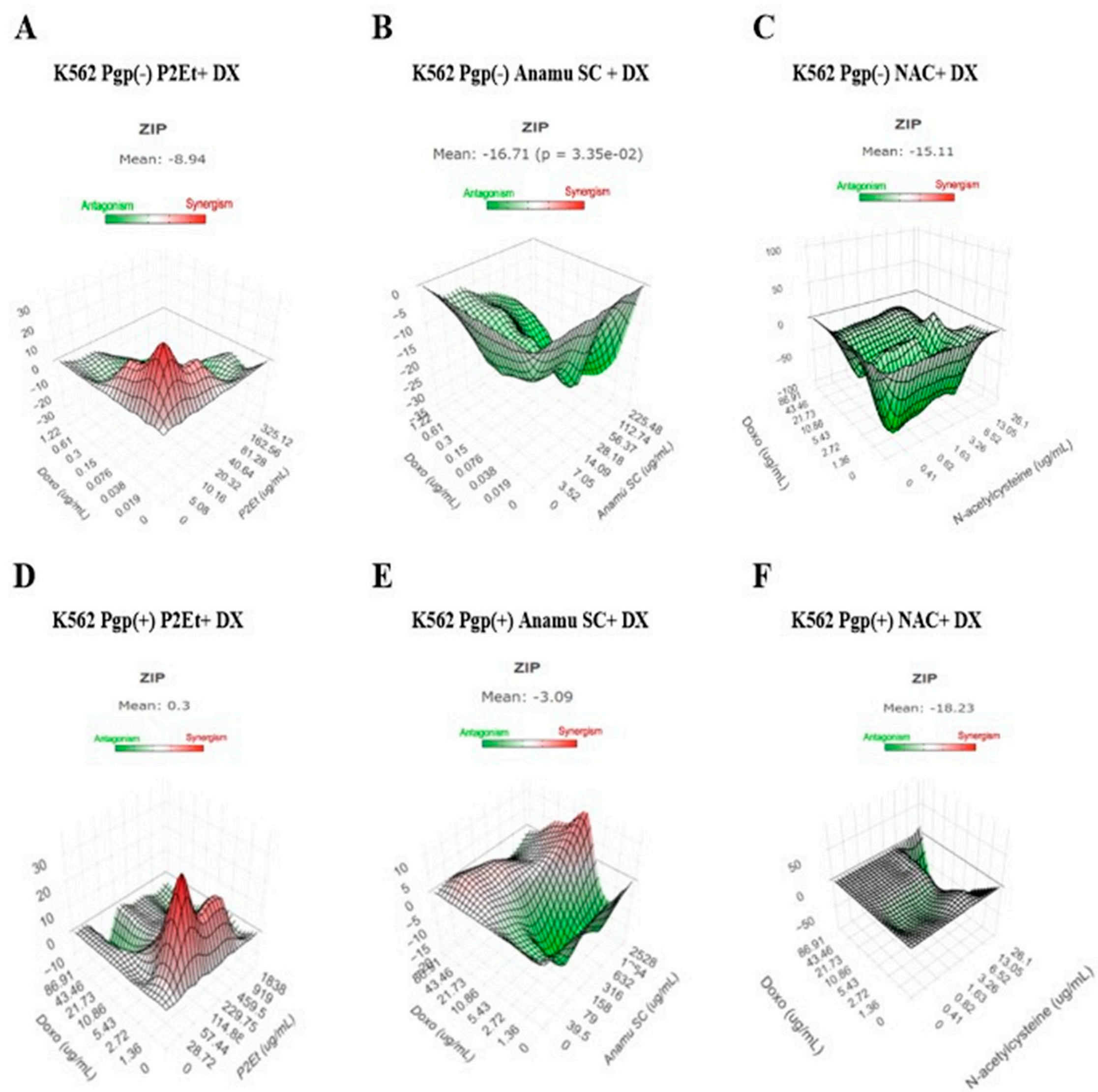

Figure S4. Effect of the combination of the P2Et and Anamu-SC extracts with doxorubicin (DX) in leukemic cells. The interaction (synergy, additivity, or antagonism) was determined using a dose-response matrix in the Synergy Finder plus

software, using the zero interaction power (ZIP) model. (**A**) Representative image of the interaction between P2Et and DX)in K562 Pgp(-) cells. (**B**) Representative image of the interaction between Anamu-SC and DX in K562 Pgp (-) cells. (**C**) Representative image of the interaction between N-acetylcysteine (NAC) and DX in K562 Pgp (-) cells. (**D**) Representative image of the interaction between P2Et and DX in K562 Pgp (+) cells. (**E**) Representative image of the interaction between Anamu-SC and DX in K562 Pgp (+) cells. (**F**) Representative image of the interaction between N-acetylcysteine (NAC) and DX in K562 Pgp(+) cells. (n=3)
